# Influence of Bone Conditions on the Accuracy of Implant Placement

**DOI:** 10.3390/bioengineering11111161

**Published:** 2024-11-18

**Authors:** Zhicheng Gong, Yuyin Shen, Shengcai Qi, Lai Cao, Xinyi Fan, Chunhui Lu, Jue Wang

**Affiliations:** 1 Department of Dental Technology, Shanghai Stomatological Hospital & School of Stomatology, Fudan University, Shanghai 200001, China; gzcisczg1982@163.com (Z.G.); yyshen01@163.com (Y.S.); harumomo1234@hotmail.com (L.C.); 2 Department of Prosthodontics, Shanghai Stomatological Hospital & School of Stomatology, Shanghai Key Laboratory of Craniomaxillofacial Development and Diseases, Fudan University, Shanghai 200001, China; qishengcai@fudan.edu.cn; 3 Department of Stomatology, Shanghai Seventh People’s Hospital, Shanghai 200137, China; f15000301486@126.com

**Keywords:** tooth-supported surgical guide, cortical bone thickness, bone density, residual ridge morphology

## Abstract

This study aimed to assess the influence of cortical bone thickness, bone density, and residual ridge morphology in the posterior mandibular area on the accuracy of implant placement using tooth-supported digital guides. The research included 75 implants from 55 patients. Each patient underwent a cone-beam computed tomography (CBCT) scan for image analysis. Simplant^®^ Pro 17 software (SIMPLANT Pro 17.01) was utilized to measure cortical bone thickness, bone density, and residual ridge morphology at the implant sites. Subsequently, 3Shape Trios software (3Shape TRIOS Design Studio 1.7.19.0) was applied to delineate optimal implant positions and design tooth-supported surgical guides. After implant treatment, the linear and angular deviations from the planned placement were quantified. Multiple linear regression, Kruskal–Wallis test, Conover–Iman test, and Bonferroni adjustment were conducted to investigate the impact of bone characteristics on implant placement precision. The tooth-supported digital guides used in this study were sufficient to fulfill the precision criteria for implant treatment. Bone density was found to significantly influence the buccal-lingual angular deviation, mesio-distal linear deviation, and mesio-distal angular deviation (*p* < 0.05). Additionally, significant variances were noted in the coronal deviation, apical deviation and depth deviation in buccal-lingual orientation, coronal deviation, and apical deviation in mesio-distal orientation across various residual ridge morphologies (*p* < 0.05). Low bone density and S-shape morphology may affect the accuracy of implant placement using tooth-supported surgical guides.

## 1. Introduction

In the realm of implant dentistry, guided by the principles of precision surgery, digital guides have been extensively utilized. They serve as repositories of essential information concerning implant placement’s direction, position, and angle. Digital guides significantly enhance dental implantations’ accuracy and predicted outcomes’ reliability [1]. As one of the categories of digital guides, tooth-supported guides are indicated for patients with partial edentulism. A systematic review indicates that tooth-supported guides exhibit higher accuracy compared to bone- and mucosa-supported guides [2]. Furthermore, they mitigate the risk of damaging vital structures such as the alveolar nerve, preventing sinus perforation, fenestration, and dehiscence [3].

The accuracy of digital guides is quantified as the lack of deviation of the actual implant position compared to the planned implant position [4]. Several factors, including the patient’s bone condition, soft tissue status, and the complexity of anatomical structure, contribute to observed deviations [5]. Among these, the bone condition predominantly influences the accuracy of implant placement by influencing the initial stability of the implant, osseointegration, and the success rate of implantation [6,7,8].

Implant placement in the mandibular posterior regions is frequently complex due to constrained spatial dimensions and limited mouth opening [9]. Besides, computed tomography scans of this region typically reveal adequate bone width but limited vertical bone height [10]. Consequently, the potential for injury to the inferior alveolar, lingual, and mental nerves are heightened [11]. Therefore, thorough assessment of bone conditions in the mandibular posterior region, coupled with the use of preoperative computer-aided planning, is imperative for the precise and secure placement of implants, as well as for the mitigation of surgical risks [12].

Several studies have analyzed the influence of bone conditions, predominantly concentrating on the impact of bone density, cortical bone thickness, and bone width on the accuracy of digitally guided surgery. Putra et al. stated that bone density significantly affects the accuracy of implant placement using computer-guided surgery [13], with higher bone density leading to better initial stability of the implant and a higher success rate of osseointegration [14,15]. The cortical bone plays an important role in bearing and dispersing stress [16]. In the mandibular posterior region, the cortical bone thickness is typically the most substantial when compared to other oral divisions [17], and this thickness significantly affects the initial stability of the implant [18,19]. Pertaining to bone width, Putra et al. measured the width 1 mm below the alveolar ridge as a benchmark for evaluation and concluded that narrow bucco-lingual width near the alveolar bone crest in the implant placement site may be a risk factor affecting the accuracy of implant placement in computer-guided surgery [13].

After tooth loss, the alveolar bone continuously undergoes horizontal and vertical bone resorption due to the loss of functional pressure stimulation. This resorption results in varying degrees of absorption, and the remaining alveolar ridge presents different morphologies [20]. Certain ridges exhibit depressions on either the buccal or lingual aspects [21], which cause changes in the bone width. Gallucci et al. have indicated that distinct morphologies of the residual ridge carry varying surgical risks and can influence the ease of implant placement [22]. Nonetheless, scant research has been dedicated to examining the potential influence of different residual ridge morphologies on the accuracy of surgical guides.

This study incorporates the morphology of the residual ridge as a critical factor in assessing bone conditions within the mandibular posterior region. The aim of this study is to evaluate the influence of cortical bone thickness, bone density, and residual ridge morphology on the accuracy of implant placement using tooth-supported surgical guides.

## 2. Materials and Methods

### 2.1. Subjects and Selection Criteria

The subjects of this study were the patients who underwent dental implant treatment in the mandibular posterior region using tooth-supported surgical guides at Shanghai Stomatological Hospital, from June 2021 to December 2023. The patients were required to have healthy local soft and hard tissues, normal mouth opening, and suitable intermaxillary distance. For better bone condition measurement, the cone-beam computer tomography (CBCT) image was required to have good visibility. Therefore, based on the selection criteria, 55 patients, with a total of 75 implants, were included. The distribution of implant dimensions and systems is represented in Table 1.

### 2.2. Clinical Procedure

Patients were subjected to radiographic examination using CBCT (NewTom, VGi, Bologna, Imola, Italy) before implant treatment. The key parameters of the device were set to: Field of view: 15 cm × 15 cm × 15 cm, voxel size: 0.3 mm, voltage: 110 kV, exposure time: 3.6 s. Intraoral scanning of the maxillomandibular dental arches and occlusal relationships was performed using a 3Shape Trios3 scanner (3Shape Trios3, Copenhagen K, Danmark) in accordance with the manufacturer’s guidelines, to obtain digital impressions. These impressions were subsequently exported in the Surface Tessellation Language (STL) format. Both CBCT data and the STL data were imported into 3Shape Trios software (3Shape Trios2021, Copenhagen K, Danmark) and Simplant^®^ Pro 17 software (Dentsply Sirona, Charlotte, NC, USA) to virtually plan the position and implant system based on the ideal prostheses and the available bone. The tooth-supported surgical guides were then designed by an experienced dental technician using 3Shape Trios software; all design parameters were set consistently. The design data were exported initially as STL files for manufacturing purposes. Additionally, the design data were saved as 3oxz files to facilitate subsequent deviation assessments.

Before initiating the implant treatment, all tooth-supported surgical guides were verified for stability and a secure fit against the remaining tooth surfaces. Subsequently, the guide was removed, and the following steps were executed in sequence: further drilling, preparation of the implant site, placement of the implant, and suturing of the surgical wound. After the implant treatment, the patient underwent CBCT examination.

### 2.3. Bone Condition Measurement

The bone density and cortical bone thickness within the implant placement area were assessed, and the residual ridge morphologies were classified utilizing the CBCT images obtained before the implant treatment. The measurement was conducted using Sploded^®^Pro 17 software.

**Figure 1:**
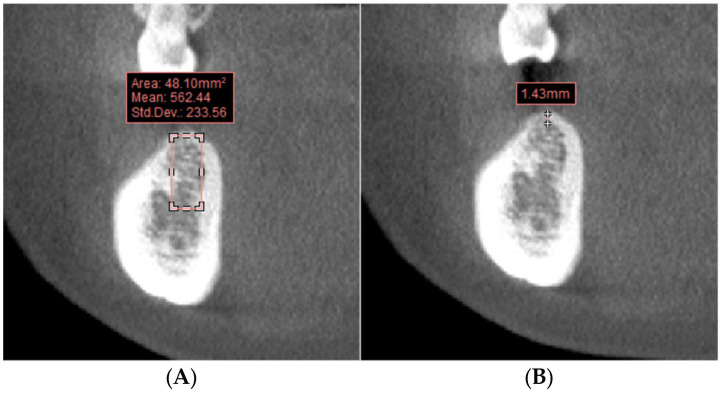
Bone condition measurement. (**A**) Bone density measurement; (**B**) cortical bone thickness measurement.

**Figure 2:**
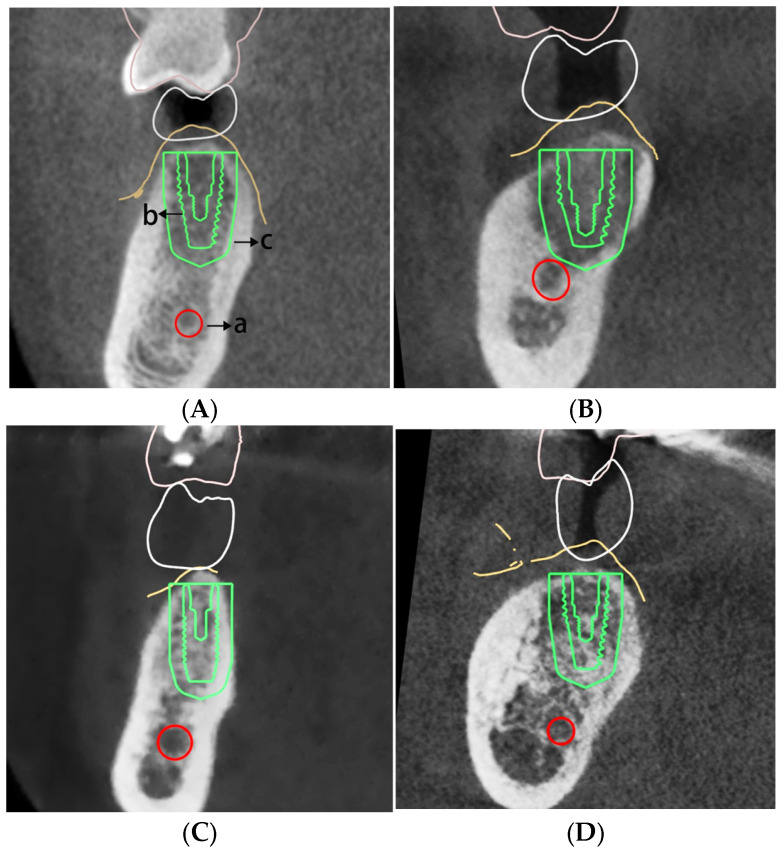
Classification of the residual ridge morphology. (**A**) Straight: the alveolar ridge and the basal bone are perpendicular to the occlusal surface, with no significant lingual concavity; (**B**) S-shape: characterized by an S-shaped alveolar ridge and basal bone, with a marked concavity on the lingual side; (**C**) Oblique: with an angled alveolar process that is narrower than the width of the basal bone; (**D**) Basal-round bone: both the buccal and lingual sides are rounded (letters on figure: a. inferior alveolar nerve; b. planned virtual implant placement; c. additional 3 mm safeguard zone designated for the implant’s protection).

In the coronal plane image of the planned implant site, a rectangle corresponding to the cross-sectional area and position of the virtual implant was delineated to measure the bone density of the implant area (Figure 1A). The cortical bone thickness was measured parallel to the central line of the virtual planned implant (Figure 1B). Classification of residual ridge morphologies was performed on the sagittal plane image at the planned implant location. As the study’s cases did not include an hourglass morphology, the residual ridge morphologies were divided into four groups: Straight (Figure 2A), S-shape (Figure 2B), Oblique (Figure 2C), and Basal-round bone (Figure 2D) [16,18]. This classification was conducted by a team of four dental technicians. In instances of disagreement regarding the classification of a residual ridge morphology, the technicians engaged in discussions to achieve a consensus.

### 2.4. Implant Deviation Measurement

**Figure 3:**
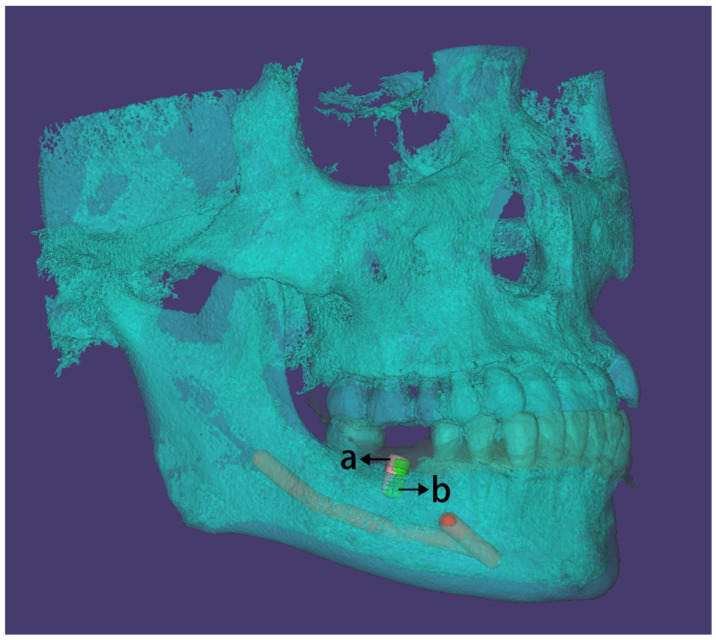
Matching digital data between the CBCT obtained before and after the implant treatment. (letters on figure: a. planned virtual implant placement; b. actual implant placement).

The 3oxz file containing the planned virtual implant and the CBCT obtained before the implant treatment, along with the CBCT obtained after the implant treatment, was imported into the 3Shape Trios software. The residual teeth served as a reference area for alignment to match the CBCT data obtained before and after the implant treatment (Figure 3).

**Figure 4:**
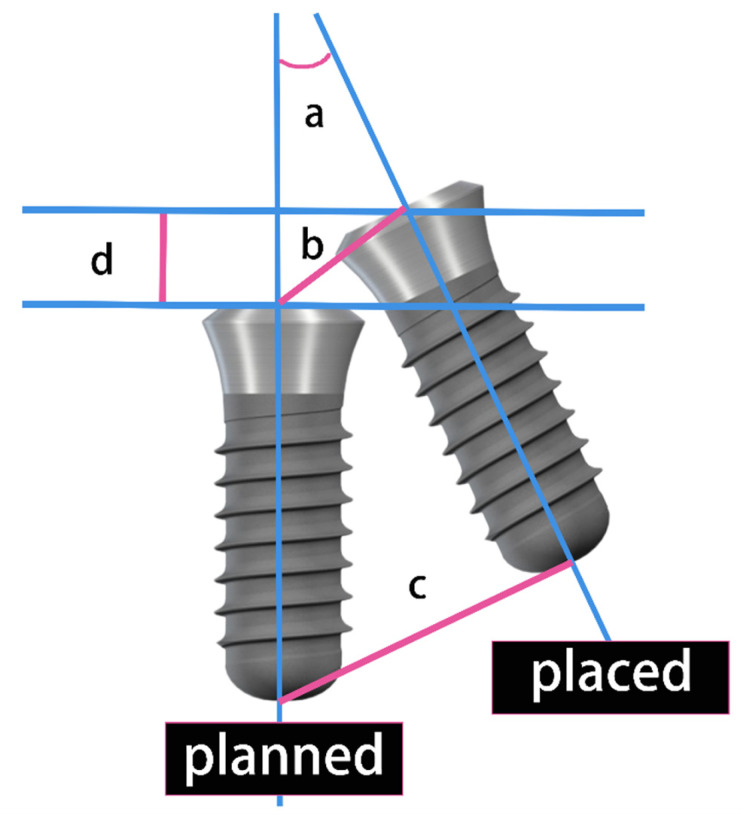
The accuracy parameter of implant placement. (a) Angular deviation; (b) coronal deviation; (c) apical deviation; (d) depth deviation.

**Figure 5:**
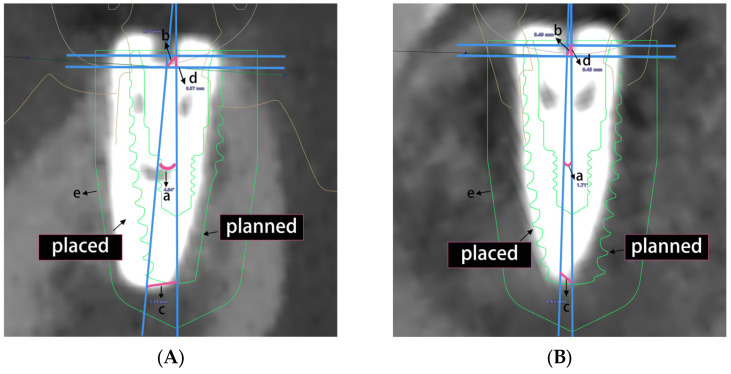
Measurement of deviations. (**A**) Buccal-lingual orientation; (**B**) mesio-distal orientation (letters on figure: a. angular deviation; b. coronal deviation; c. apical deviation; d. depth deviation; e. additional 3 mm safeguard zone designated for the implant’s protection).

In the buccal-lingual and mesio-distal orientations, the three-dimensional axis of observation was adjusted to align the sagittal axis with the mesio-distal orientation of the alveolar ridge at the implant site. The sagittal section at this angle was designated for mesio-distal measurements, while the coronal section was used for buccal-lingual measurements. On these sections, the long axis midline, platform midpoint, and apex point of both the planned virtual implant and the actual implant placement were identified. The accuracy of implant placement was assessed in both the buccal-lingual and mesio-distal directions using the following parameters (Figure 4). The measurement of deviations is shown in Figure 5. To ensure measurement precision, each case was evaluated for deviations thrice, with the mean value being recorded as the definitive deviation.

### 2.5. Statistical Analysis

Statistical analysis was conducted using the IBM SPSS Statistics 25.0 software (IBM SPSS Statistics for Windows version 25.0, IBM Corp, Armonk, NY, USA). A multiple linear regression analysis was conducted to examine the relationship between combined bone conditions and the accuracy parameters of implant placement. Independent variables included bone density and cortical bone thickness, while dependent variables comprised angular deviation, coronal deviation, apical deviation, and depth deviation in both buccal-lingual and mesio-distal directions. Kruskal-Wallis test was applied to assess the impact of residual ridge morphology on implant placement accuracy. Post-hoc tests were conducted using Conover-Iman test, and *p*-values were adjusted by Bonferroni adjustment. A *p*-value less than 0.05 was considered significant.

## 3. Results

In this retrospective study, 55 patients, ranging in age from 25 to 73, consisting of 23 males and 32 females, with a total of 75 implants, were included. Fifteen mandibular premolar and sixty mandibular molar implant locations were analyzed. Fourty-nine implant sites had a distal tooth, and twenty-six sites did not.

### 3.1. Overall Accuracy of Implant Placement

This study included a total of 75 implants in the mandibular posterior region. The overall three-dimensional deviations of implants guided by tooth-supported surgical guides are presented in Table 2.

### 3.2. Influence of Bone Density and Cortical Bone Thickness on the Accuracy of Implant Placement

The result of multiple regression analysis of implant placement accuracy using bone density and cortical bone thickness as dependent variables are shown in Table 3. Bone density was found to significantly influence the accuracy of implant placement regarding the angular deviation in buccal-lingual orientation, and the angular deviation and the apical deviation in the mesio-distal orientation (*p* < 0.05). Conversely, the effect of cortical bone thickness on implant placement accuracy was not statistically significant (*p* > 0.05).

**Figure 6:**
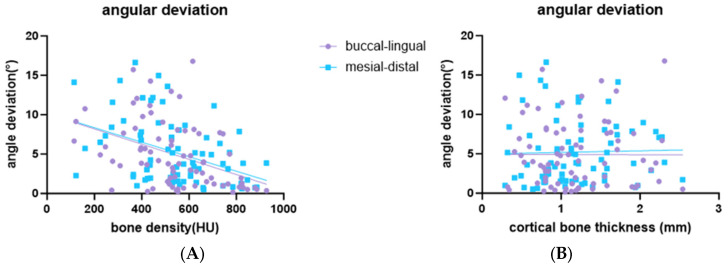
Scatter plot showing a linear correlation between bone condition and angular deviation. (**A**) Linear correlation between bone density and angular deviation; (**B**) linear correlation between cortical bone thickness and angular deviation.

**Figure 7:**
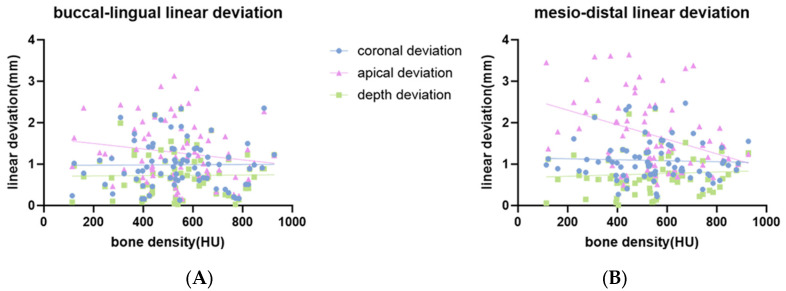
Scatter plot showing a linear correlation between bone density and linear deviation. (**A**) Buccal-lingual linear deviation; (**B**) mesio-distal linear deviation.

**Figure 8:**
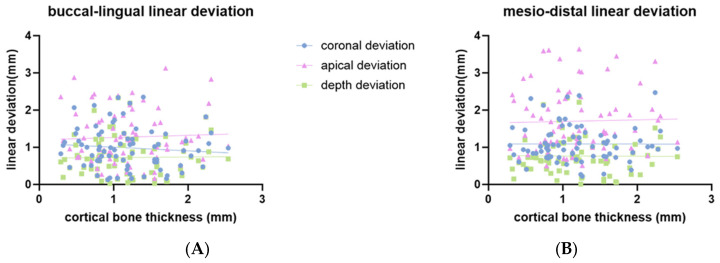
Scatter plot showing a linear correlation between cortical bone thickness and linear deviation. (**A**) Buccal-lingual linear deviation; (**B**) mesio-distal linear deviation.

The distribution of placement accuracy parameters related to bone conditions (bone density and cortical bone thickness) is shown in Figure 6, Figure 7 and Figure 8. There were significant negative correlations between bone density and buccal-lingual angular deviation (r = −0.44; *p* < 0.001), mesio-distal angular deviation (r = −0.44; *p* < 0.001) (Figure 6A) and mesio-distal apical deviation (r = −0.4; *p* < 0.001) (Figure 7B). There was no statistically significant correlation for bone density with buccal-lingual coronal, apical, and depth linear deviation (Figure 7A). There was no statistically significant correlation for cortical bone thickness with any deviation parameters (Figure 6B) and Figure 8.

### 3.3. Influence of Residual Ridge Morphology on the Accuracy of Implant Placement

Kruskal–Wallis test of the three-dimensional deviations of implants with different residual ridge morphologies is presented in Table 4. Significant differences in linear deviations were observed among different residual ridge morphologies.

In buccal-lingual orientation, for coronal deviation, there were significant differences between Straight morphology (A) and S-shape morphology (B), Oblique morphology (C), and Basal-round morphology (D) (*p* = 0.02). The differences between A and B,C,D remain significant after Bonferroni correction (*p* < 0.001; *p* = 0.002; *p* = 0.006, respectively). For apical deviation, there were significant differences between A and B,C,D (*p* = 0.011). The differences between A and B,C,D remain significant after Bonferroni correction (*p* < 0.001). For depth deviation, there were significant differences between A and B,C (*p* = 0.02). The differences between A and B,C remain significant after Bonferroni correction (*p* < 0.001; *p* = 0.002, respectively). For angular deviation, there were no significant differences between each morphology.

In mesio-distal orientation, for coronal deviation, there were significant differences between A and B,C,D (*p* = 0.005). The differences between A and B,C,D remain significant after Bonferroni correction (*p* < 0.001). For apical deviation, there were significant differences between A and B,C,D (*p* = 0.001). The differences between A and B, C, D remain significant after Bonferroni correction (*p* < 0.001). For angular and depth deviation, there were no significant differences between each morphology.

## 4. Discussion

Prior to the implant treatment, assessing bone conditions helps formulate a more precise treatment protocol, including determining the optimal implant position, angle, and depth, as well as the need for bone augmentation surgery [23]. Numerous studies emphasize the significance of measuring bone density before implant planning [24,25]. Putra et al. also advocate for including cortical bone thickness as a factor in these assessments [13]. The diagnostic tool employed to evaluate bone condition in this study is CBCT. Although pixel or voxel values derived from CBCT may not be absolute [23], existing research substantiates the feasibility of CBCT for assessing the bone density and cortical bone thickness in the implant region [26,27].

The three-dimensional deviation measurements of the implants in this study align with the findings reported by Schneider et al. [28]. The deviations fall within the acceptable range, with an average linear deviation of less than 2 mm and an angle deviation less than 8°, as proposed by Yiting Shi et al. [1]. These parameters are deemed sufficient to fulfill the precision criteria for implant treatment.

The findings from the multiple regression analysis revealed no significant correlation between cortical bone thickness and implant placement accuracy using tooth-supported surgical guides, which is consistent with the research of Putra et al. [13]. This outcome may be attributed to the substantial cancellous bone area surrounding the cortical bone, which interacts with the cortical bone and exerts a significant biomechanical influence during the implant load-bearing process. According to Kinoshita et al., in jawbones with sufficient cortical bone, the cortical bone primarily manages the main biomechanical effect, while the cancellous bone serves as a supplementary load-transfer path. However, when the cortical bone is thinner, the cancellous bone compensates by distributing stress to the surrounding cortical bone [17]. This study did not consider the cancellous bone as a factor. Bone density demonstrates a significant negative correlation with the accuracy of implant placement assisted by tooth-supported surgical guides. At a low bone density level, implant deviation, particularly angular deviation, will become higher. This result is consistent with the previous conclusion that lower bone density values corresponds to greater angular deviation [15]. The rationale may lie in the reduced resistance of spongy bone to deviation during implant site preparation, which could cause the drill to stray from the intended path. Nonetheless, the r^2^ value reveals that, in our highest significant model, only 19% of implant deviation is attributable to bone conditions. This suggests the presence of potential confounding factors, such as guide manufacturing precision, data acquisition methods, guide planning software, surgical techniques, and complex implant surgery [25,29], which were not factored into the measurements.

This study identified that the straight residual ridge morphology exhibited reduced coronal, apical, and depth deviations in the buccal-lingual direction relative to other morphologies. In the mesio-distal direction, both coronal and apical deviations were also found to be reduced compared to other categories. This may be attributed to the lack of significant curvature or inclination of the straight morphology, which presents a straight and relatively flat configuration. Consequently, for this type of morphology, non-angled implants are typically applicable, given the adequate bone height and lower implant complexity [22]. The S-shape morphology demonstrated the highest deviations, potentially due to its curvature, which may predispose the drill to slippage. Consequently, caution is warranted when encountering this type of morphology. Therefore, experienced clinicians should undertake the selection and execution of a suitable treatment protocol [20]. The oblique morphology, characterized by a narrow upper alveolar ridge width, may result in initial drill point deviation from the planned position, causing increased implant placement deviations [13]. Hence, the utilization of a full-arch guide is recommended to minimize these deviations. For basal-round bone, special attention to remaining bone height is necessary to select appropriate implants and avoid damage to critical structures, such as nerves. The variability in residual ridge morphology is predominantly attributed to post-extraction bone resorption and remodeling [22]. Gender, age, and genetics are factors that influence alveolar bone resorption and healing [30]. Lifestyle choices such as diet, exercise habits, and smoking also play a crucial role in this process [31]. Furthermore, the tooth extraction technique can influence the healing and resorption processes of the alveolar bone [32].

The findings of this study must be considered in light of some limitations. Firstly, cortical bone thickness and bone density were only measured on the coronal plane image at the planned implant site, a method that may not accurately reflect the three-dimensional interactions between the bone and the implant. Secondly, previous research has suggested that Black patients may exhibit lower alveolar bone density compared to their White counterparts [33]. Such a finding indicates that ethnicity may be one of the significant variables influencing the alveolar bone condition of patients. Therefore, the exclusion of the Asian population from this research limits the comprehensiveness of the results. Future investigations should explore the impact of bone conditions on implant deviation across various ethnic groups. Thirdly, in the posterior mandibular region, implant surgery often encounters spatial challenges due to limitations imposed by the degree of mouth opening and the confined surgical area [34]. Such constraints can lead to deviations in implant placement, as the restricted space hinders the surgeon’s ability to manipulate the surgical guide effectively. Additionally, due to the limited total sample size within the specified range, this study did not control for variables such as the number of missing teeth, tooth position, the presence or absence of distal-extension dentition defects, implant dimensions and systems, and the operating surgeon. No further analysis was performed to investigate the correlation between deviations. Future research is anticipated to exert greater control over these potential confounding factors, thereby ensuring a more homogeneous selection of research subjects for measurement and statistical analysis.

## 5. Conclusions

Bone conditions can affect the accuracy of implant placement when using tooth-supported surgical guides in implant treatment. An implant placement site with low bone density and/or S-shape morphology may lead to deviations in implant placement.

## Figures and Tables

**Table 1:** The distribution of implant dimensions and systems.

Implant System	Implant Dimension (Diameter × Length)	Total Implants
Alpha-Bio Tec, ICE (Warsaw, Poland)	4.65 mm × 11.5 mm	1
DENTSPLY Implants, OsseoSpeed™ TX (York, PA, USA)	4.0 mm × 11.0 mm	2
Nobel Biocare, NobelActive^®^, RP (Gothenburg, Sweden)	4.3 mm × 10.0 mm	1
Nobel Replace^®^, Conical Connection (Gothenburg, Sweden)	4.3 mm × 10.0 mm	4
	4.3 mm × 11.5 mm	1
	5.0 mm × 8.0 mm	1
	5.0 mm × 10.0 mm	2
	5.0 mm × 11.5 mm	1
Straumann BLT, Roxolid^®^, Loxim^®^ (Basel, Switzerland)	3.3 mm × 12.0 mm	1
	4.1 mm × 10.0 mm	12
	4.1 mm × 12.0 mm	1
	4.8 mm × 8.0 mm	12
	4.8 mm × 10.0 mm	29
	4.8 mm × 12.0 mm	2
Straumann BL, Roxolid^®^, Loxim^®^ (Basel, Switzerland)	4.8 mm × 8.0 mm	2
	4.8 mm × 10.0 mm	3

**Table 2:** Overall three-dimensional deviations of implants guided by tooth-supported surgical guides (mean ± SD *n* = 75) (depth is represented by absolute value).

	Angular Deviation (°)	Coronal Deviation (mm)	Apical Deviation (mm)	Depth Deviation (mm)
Buccal-lingual orientation	4.90 ± 4.15	0.99 ± 0.55	1.27 ± 0.71	0.73 ± 0.53
Mesio-distal orientation	5.21 ± 4.12	1.09 ± 0.48	1.70 ± 0.85	0.77 ± 0.49

**Table 3:** Multiple linear regression analysis of the correlation between bone density and cortical bone thickness, with accuracy parameters of implant placement (depth is represented by absolute value).

		Buccal-Lingual Orientation				Mesio-Distal Orientation			
		Angular Deviation	Coronal Deviation	Apical Deviation	Depth Deviation	Angular Deviation	Coronal Deviation	Apical Deviation	Depth Deviation
Bone density	β	−0.01 ***	0.355	−0.658	0.038	−0.009 ***	−0.117	−1.759 ***	0.173
	95% CI	[−0.014, −0.004]	[−0.68, 0.735]	[−1.554, 0.238]	[−0.634, 0.709]	[−0.014, 0.004]	[−0.734, 0.5]	[−2.756, −0.761]	[−0.448, 0.793]
Cortical bone thickness	β	−0.026	−0.098	0.06	0.014	0.202	−0.001	0.042	−0.008
	95% CI	[−1.845, 1.792]	[−0.341, 0.144]	[−0.253, 0.372]	[−0.216, 0.246]	[−1.605, 2.008]	[−0.213, 0.211]	[−0.328, 0.413]	[−0.222, 0.206]
r^2^		0.19	0.01	0.04	0.00	0.19	0.00	0.16	0.01
Overall *p*-value		<0.001	0.69	0.26	0.99	0.001	0.93	0.002	0.84
*** *p* < 0.001									

**Table 4:** Kruskal–Wallis test of the three-dimensional deviations of implants with different residual ridge morphologies. (A: Straight; B: S-shape; C: Oblique; D: Basal-round bone).

		Residual Ridge Morphology	n	Rank Mean	H	*p*	Conover–Iman Post hoc	Bonferroni Correction
Buccal-lingual orientation	angular deviation	A	21	31.05	6.013	0.111		
		B	20	47.45				
		C	17	36.88				
		D	17	36.59				
	coronal deviation	A	21	26.1	9.785	0.02	A < B ** A < C ** A < D *	A < B *** A < C ** A < D **
		B	20	46.18				
		C	17	42.38				
		D	17	38.71				
	apical deviation	A	21	24.67	11.105	0.011	A < B ** A < C ** A < D **	A < B *** A < C *** A < D ***
		B	20	44.85				
		C	17	42.44				
		D	17	41.97				
	depth deviation	A	21	27.38	9.854	0.02	A < B ** A < C **	A < B *** A < C **
		B	20	46.43				
		C	17	44.47				
		D	17	34.74				
Mesio-distal orientation	angular deviation	A	21	28.98	5.962	0.116		
		B	20	44.05				
		C	17	42.74				
		D	17	37.29				
	coronal deviation	A	21	23.71	12.656	0.005	A < B *** A < C ** A < D **	A < B *** A < C *** A < D ***
		B	20	44.88				
		C	17	43.12				
		D	17	42.44				
	apical deviation	A	21	22.14	16.043	0.001	A < B *** A < C *** A < D ***	A < B *** A < C *** A < D ***
		B	20	44.18				
		C	17	47.06				
		D	17	41.26				
	depth deviation	A	21	32.79	1.7	0.637		
		B	20	40.67				
		C	17	39.47				
		D	17	39.82				
* *p* < 0.05, ** *p* < 0.01, *** *p* < 0.001								

## Data Availability

The raw data supporting the conclusions of this article will be made available by the authors on request.
